# Short implants (<8mm) versus longer implants (≥8mm) with lateral sinus floor augmentation in posterior atrophic maxilla: A meta-analysis of RCT`s in humans

**DOI:** 10.4317/medoral.23248

**Published:** 2020-02-10

**Authors:** Naroa Lozano-Carrascal, Albert Anglada-Bosqued, Oscar Salomó-Coll, Federico Hernández-Alfaro, Hom Lay Wang, Jordi Gargallo-Albiol

**Affiliations:** 1 Assistant Professor. International Master’s Degree in Oral Surgery. Oral and Maxillo-facial Surgery Department. International University of Catalonia. Barcelona, Spain; 2 nternationa Master’s Degree in Oral Surgery Student. International University of Catalonia. Oral and Maxillo-facial Surgery Department. Barcelona, Spain; 3 Professor and Chairman of the Department of Oral and Maxillofacial Surgery. International University of Catalonia, Barcelona, Spain; 4 Professor and Director of Graduate Periodontics, Department of Periodontics and Oral Medicine. University of Michigan, School of Dentistry. Michigan. USA; 5 Associate Professor and Director of International Master in Oral Surgery. Oral and Maxillo-facial Surgery Department. International University of Catalonia, Barcelona, Spain

## Abstract

**Background:**

One of the greatest challenges that dentists face today is to rehabilitate severe atrophied alveolar ridges in partially and completely edentulous patients with implants. Despite the high survival rate of implants placed next to sinus elevation, this technique presents complications that can be avoided by placing short implants, an option that also presents high survival rates. For this reason, the aim of this study is to compare the survival rate, marginal bone loss and complications associated with short implants (<8 mm) versus longer implants (≥8mm) placed with lateral sinus floor elevation in posterior atrophic maxillae.

**Material and Methods:**

A literature search was conducted by two independent reviewers in the PubMed/Medline (National Library of Medicine, Washington, DC) electronic database for articles published from January 2007 to July 2018. Seven qualified articles were selected for the meta-analysis.

**Results:**

The test for overall effect did not find statistical significance in the survival rates, overall complications, intra-operative complications, post-operative complications and prosthetic complications. However, the test showed statistically significant differences in biological complications in favor of standard implants, and marginal bone loss between control and test groups in favor of short implants (<8mm) was found.

**Conclusions:**

Within the limitations of the present study, prosthetic rehabilitations with short implants (<8mm) in posterior maxilla is a reliable treatment option as an alternative to lateral wall sinus floor augmentation.

** Key words:**Short implant, lateral sinus floor augmentation, Randomized controlled trial, Survival rate, Complications, Marginal bone loss.

## Introduction

One of the greatest challenges that dentists face today is to rehabilitate severe atrophied alveolar ridges in partially and completely edentulous patients with implants. Following tooth loss, jaws undergo vertical collapse due to increased osteoclast activity, which takes place in response to the absence of functional load transmission to the alveolar bone. Bone resortion is aggravated by the physiological process of sinus pneumatization especially in the maxillary posterior area (1). Therefore, bone quantity and quality is often insufficient for the ideal three-dimensional (3D) implant positioning. Several bone augmentation techniques have been proposed to overcome these problems. Among these, Sinus floor elevation is considered to be the most reliable surgical technique for increasing bone height in the posterior maxilla (2). Two sinus floor elevation techniques have been described by Wang *et al*. [2008] (3): lateral approach (LSFE), when the residual bone volume is less than ≤5 mm, or crestal (CSFE) approach when residual bone height is more than 6 mm. Both techniques have reported Research reports high survival rates, 100% after 5-year follow-up (4) and 97% after a 10-year follow-up (5) and success rates 98% after a 3-year follow-up (6).

Due to the high percentage of anatomical variations among patients (7) and the sensitivity of the technique, these procedures are not exempt from complications: Schneiderian membrane perforation, sinusitis, nasal bleeding, hematomas, post-operative pain, dehiscence, graft failure, or migration of the implant into the sinus cavity are common complications associated with sinus floor elevation surgery (8,9). For this reason, several alternatives have been proposed to avoid sinus lifting, such as tilted implants or short implants (10).

Recently, implants as short as 8 mm have been considered as standard implants in several published articles (11). Short implants are slowly being accepted by patients and clinicians because they are associated with a less invasive procedure, leading to a smaller scale intervention, shorter intra-operative time, less morbidity, and lower treatment cost (12). Traditionally, short implants have been related to lower survival rates and unpredicTable outcomes. But more recently, technical and manufacturing developments have improved implant surfaces and connections and nowadays short implants have a failure rate of under 4% for ≤8mm implants, a failure rate similar to longer implants (13).

The aim of the present study was to compare the survival rate, marginal bone loss and complications associated with short implants (<8 mm) versus longer implants (≥8mm) placed with lateral sinus floor elevation in posterior atrophic maxillae.

## Material and Methods

- Study Registration

This systematic review and meta-analysis has been registered in the Prospero Database (International prospective register of systematic reviews - https://www.crd.york.ac.uk/prospero/) under the tittle: “Short implants (<8mm) versus longer implants (≥8mm) with lateral sinus floor augmentation in posterior atrophic maxilla: A meta-analysis of RCT`s in humans” (ID:92413).

- Focused Question 

Following Preferred Reporting Items for Systematic Reviews and Meta-Analyses (PRISMA) guidelines (14), a specific answerable question was formulated according to PICO(S) recommendations (Participants, Interventions, Control, Outcomes, Study):

(P) Participants: Patients who received at least one dental implant in the posterior area of the upper maxilla.

(I) Type of intervention: at least one short dental implant placement (<8mm) in the posterior area of the upper maxilla without lateral sinus floor elevation procedure.

(C) Control intervention: at least one long dental implant (≥8mm) placed simultaneously or deferred with sinus membrane elevation via lateral sinus floor elevation procedure.

(O) Outcome measures: implant survival rate, intra- and post-operative surgical complications, biological and prosthetic post-operative complications, marginal bone loss (MBL).

(S) Study type: randomized controlled clinical trials (RCTs).

- Search Strategy

A literature search was conducted by two independent reviewers (LN and AA) in the MEDLINE, EMBASE, Cochrane Central Register of Controlled Trials, and Cochrane Oral Health Group Trials Register databases.

The research included references up to July 2018, using different combinations and Boolean Operators (AND, OR, NOT) with the following search terms/key words: “short implants”, “longer implant”, “standard implant”, “bone augmentation”, “sinus lift”, “randomized control trial”, “atrophic maxilla”, “posterior maxilla”.

Following the electronic search, a further manual search was performed in the websites of the leading scientific journals on dentistry and implant dentistry.

Crossed-references were screened to identify other potentially relevant articles.

- Eligibility criteria 

Studies were deemed eligible if they met the following criteria: 1) Human subjects with posterior maxilla atrophy; 2) Randomized Clinical Trials (RCT); 3) the presence of a study group (receiving one or more short implant (<8 mm) and a control group (receiving long implants [≥8mm] simultaneously or deferred with lateral sinus floor elevation); 4) studies with a minimum follow-up period of >12 months after prosthesis placement; 5) results providing data on survival rates, complications, and marginal bone loss; 6) articles published in English. Exclusion criteria comprised of: 1) animal studies; 2) human studies with less than 15 subjects with posterior maxilla atrophy; 3) studies with a follow-up of <12 months after prosthetic loading; 4) prospective cohort studies, case reports, case series, retrospective studies, systematic reviews; and 5) articles that failed to provide sufficient information.

- Data extraction

The following information was extracted from the publications included for analysis: 1) author and year of publication; 2) duration of follow-up; 3) patient and implant sample; 4) systemic, periodontal, and smoking status; 5) time of loading; 6) implant location; 7) setting and funding; 8) preoperative preparation; 9) treatment control group; 10) treatment study group; 11) residual bone height; 12) post-surgical instructions; 13) augmentation technique; 14) survival rate; 15) intra/post-operative and biological/prosthetic complications; 16) marginal bone loss (MBL); and 17) study conclusions as reported by the authors.

Two reviewers (LN and AA) carried out the selection process, screening the articles’ titles and abstracts. The full texts of all studies of possible relevance were then obtained, and eligibility assessment and data extraction were performed independently in an un-blinded standardized manner by the two authors; any disagreement between the reviewers was resolved through discussion. When the reviewers did not agree, a third reviewer (SO) analyzed the text to decide whether the article should be included or excluded.

- Quality Assessment

The reviewers A.A and N.L assessed the quality of each study independently. Disagreements on validity assessment were resolved by consensus and discussion; when consensus could not be reached, a third reviewer was consulted (JG). The methodological quality of the RCT`s were assessed using the Cochrane Risk of Bias Tool for Randomized Controlled Trials (Table 1).

- Statistical Analysis

Statistical analysis was performed using R Project software© (The R Foundation, Bell Laboratories, formerly AT&T, now Lucent Technologies by John Chambers and Colleagues). The Chi2 test was used to evaluate heterogeneity across the studies; subgroup analysis was performed when heterogeneity was significant (*p-value* <0.05) and the I2 statistic expressed the percentage of heterogeneity, with 25% corresponding to low heterogeneity, 50% to moderate, and 75% to high. A test of overall effects was used to evaluate significance between the groups; a *p-value* of less than 0.05 was considered statistically significant. A forest plot was drawn to represent estimates of relative effect, expressed as risk ratio (RR) with a 95% confidence interval (CI).

## Results

- Study selection

**Figure 1:**
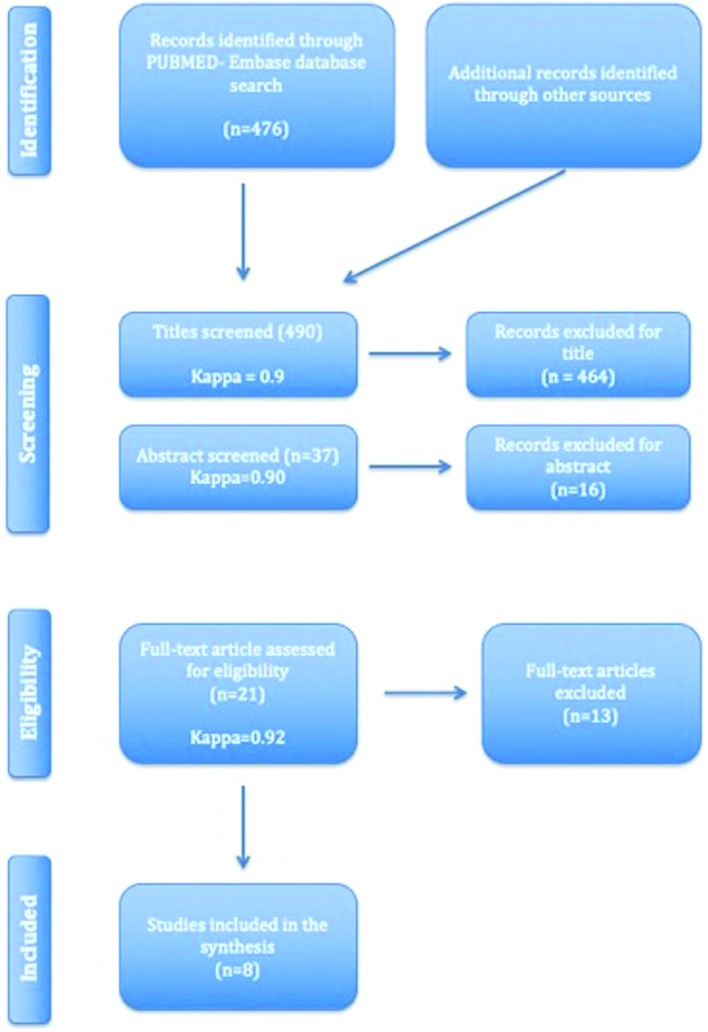
PRISMA flowchart of the screening process in different databases.

Search results based on the PRISMA guidelines are depicted in Fig. 1. The initial search identified 482 titles, 476 PubMed Embase Database and 6 additional records identified through hand-searching. After elimination by screening all titles and abstracts, twenty-one studies were left for full-text assessment. After full-text screening, 14 articles were excluded due to failed to meet the inclusion criteria (Table 1) (15-29), leaving a total of 8 RTCs (30-37) for inclusion in the meta-analysis (Table 2).

**Table 1:**
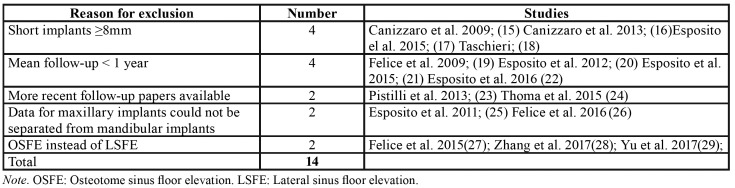
Excluded Studies.

**Table 2:**
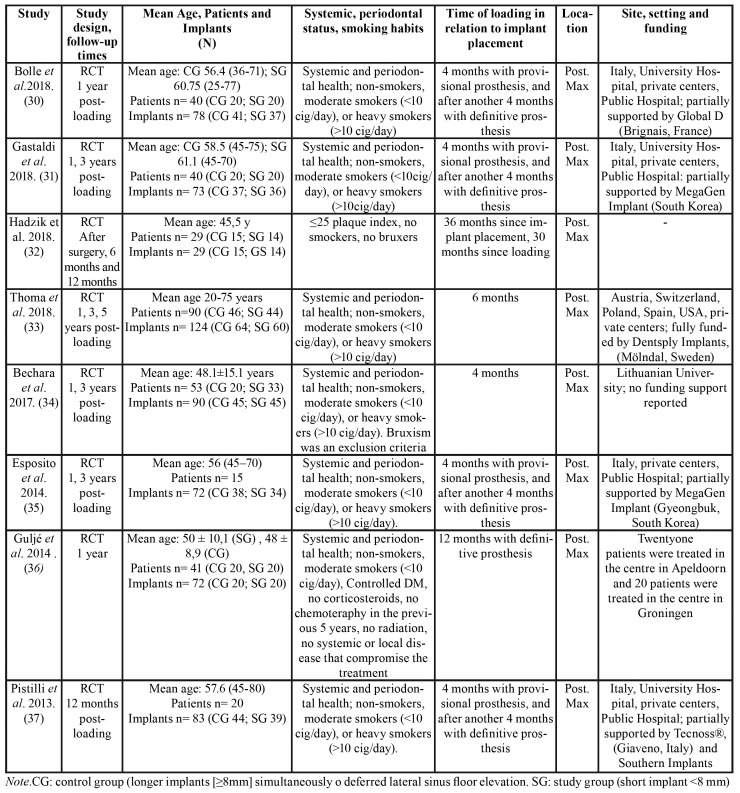
General overview of the studies included for analysis.

The selection of RCTs included a total of 328 patients (although Esposito *et al*. (35) and *Pi*stilli *et al*.(37) did not reported the number of patients in each group), with a total of 621 implants (296 allocated to study group, and 316 to control group). Six studies only investigated partially edentulous patients (30-33,35,37); only two studies employed a split-mouth design (34,35), while the rest had parallel treatment arms (Table 3).

- Quality assessment 

Table 4 summarizes the results of bias risk assessment in the included RCTs and The Cochrane Risk of Bias Tool for Randomized Controlled Trials criteria.

**Table 3:**
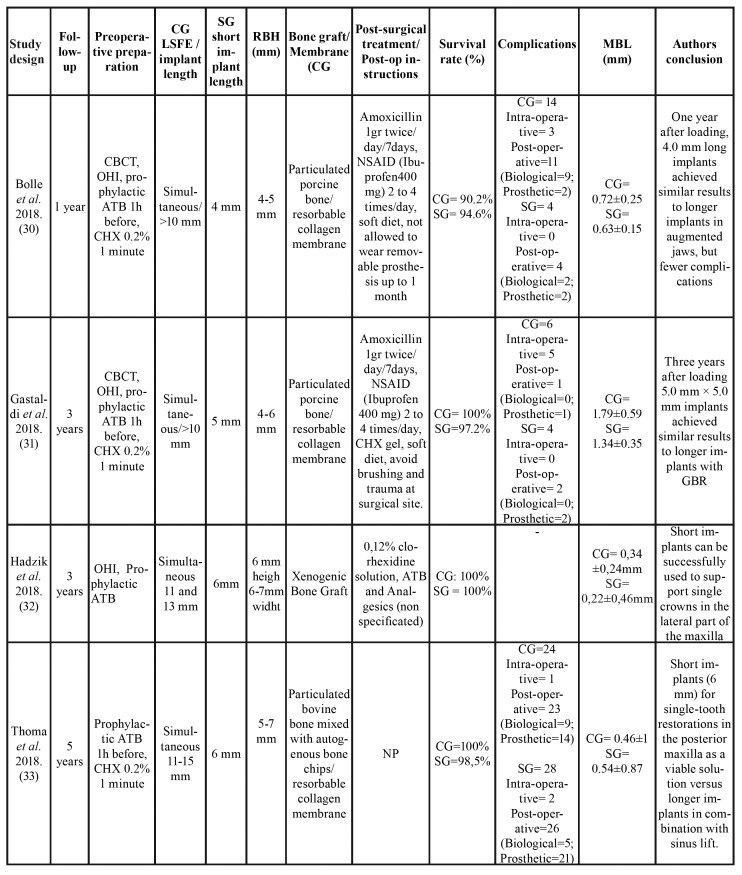
General characteristics of the intervention and results.

**Table 3 cont.**
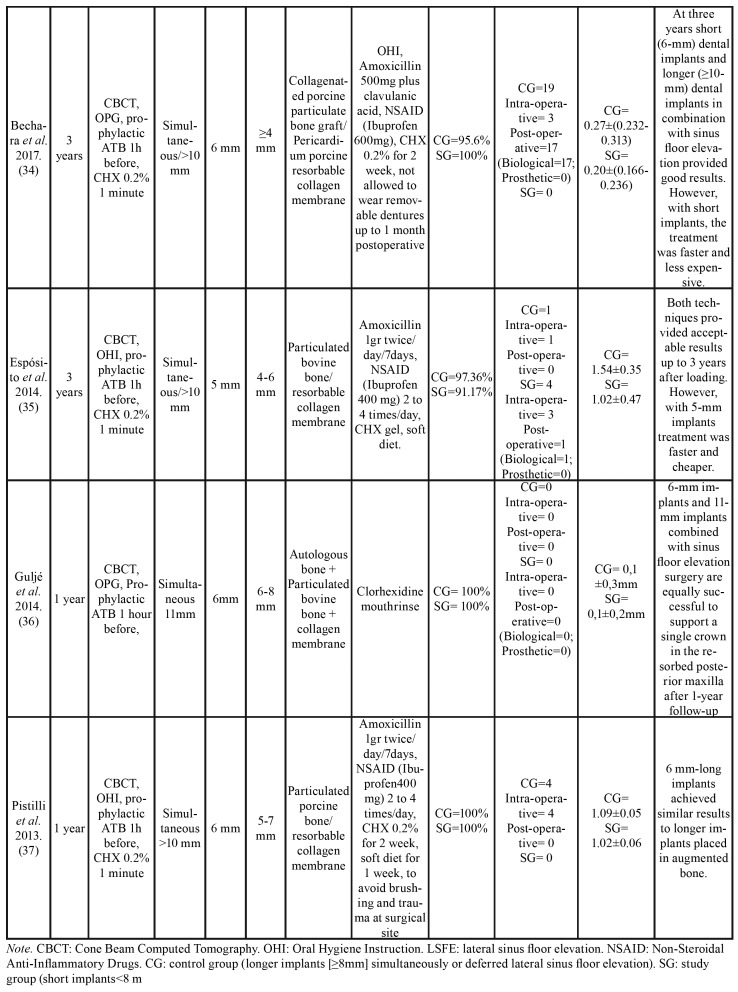
General characteristics of the intervention and results.

**Table 4:**
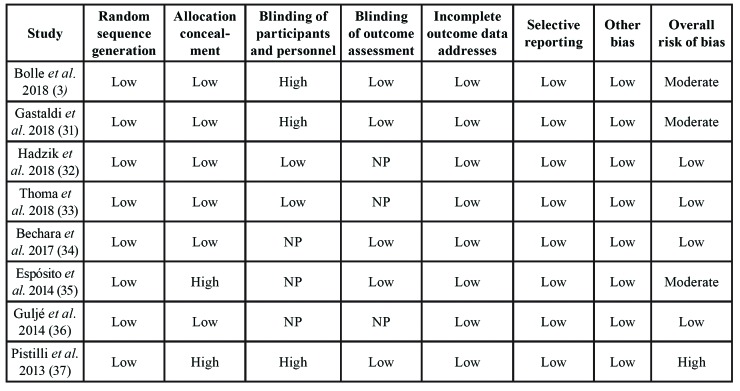
Bias risk assessment for the included RCTs using The Cochrane Risk of Bias Tool for Randomized Controlled Trials.

- Survival rate

**Figure 2:**
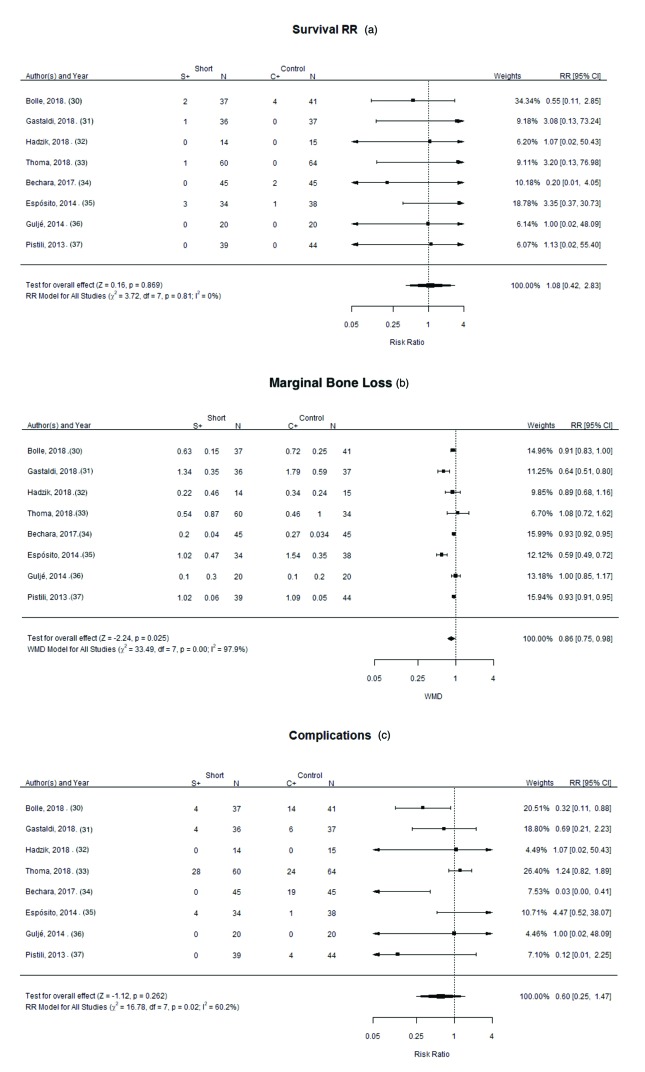
Forest plot for the event: (a) “implant survival rate; (b) “marginal bone loss” ”; (c) “complications”.

A random-effects model was used to assess the survival rate of implants; statistically significant heterogeneity was not found among the publications (I2=0%; *p*=0.81). The test for overall effect showed no statistically significant differences in the survival rate of short implants (<8mm) compared to longer implants (≥8mm) with lateral sinus floor elevation (Risk Ratio [RR] of 01.08; 95% CI: [0.42-2.83]; *p*= 0.8) (Fig. 2).

- Marginal Bone Loss

The random-effect model showed highly significant heterogeneity between the studies (I2=97.9%; *p*=0.00). The overall effect test showed statistically significant differences in marginal bone loss between control and study groups (*p*=0.026). A RR of 0.86; 95% CI: [0.75, 0.98] in favor of short implants (<8mm) (Fig. 2). This finding implies that the risk of marginal bone loss in patients receiving longer implants (≥8mm) with lateral sinus floor elevation is significantly higher than patients receiving short implants (<8mm). However, these results should be treated with caution due to the different follow-up periods among the studies analyzed.

- Complications

The test for overall effect did not find statistical significance (RR of 0.60; 95% CI: [0.25-1.47]; *p*=0.262). For this variable the random-effects model showed a statistically significant heterogeneity between the studies (I2=60,2%; *p*=0.03) (Fig. 2). So, complications were divided into four groups: (3a) intra-operative complications; (3b) post-operative complications: (3c) biological complications; and (3d) prosthetic complications.

**Figure 3:**
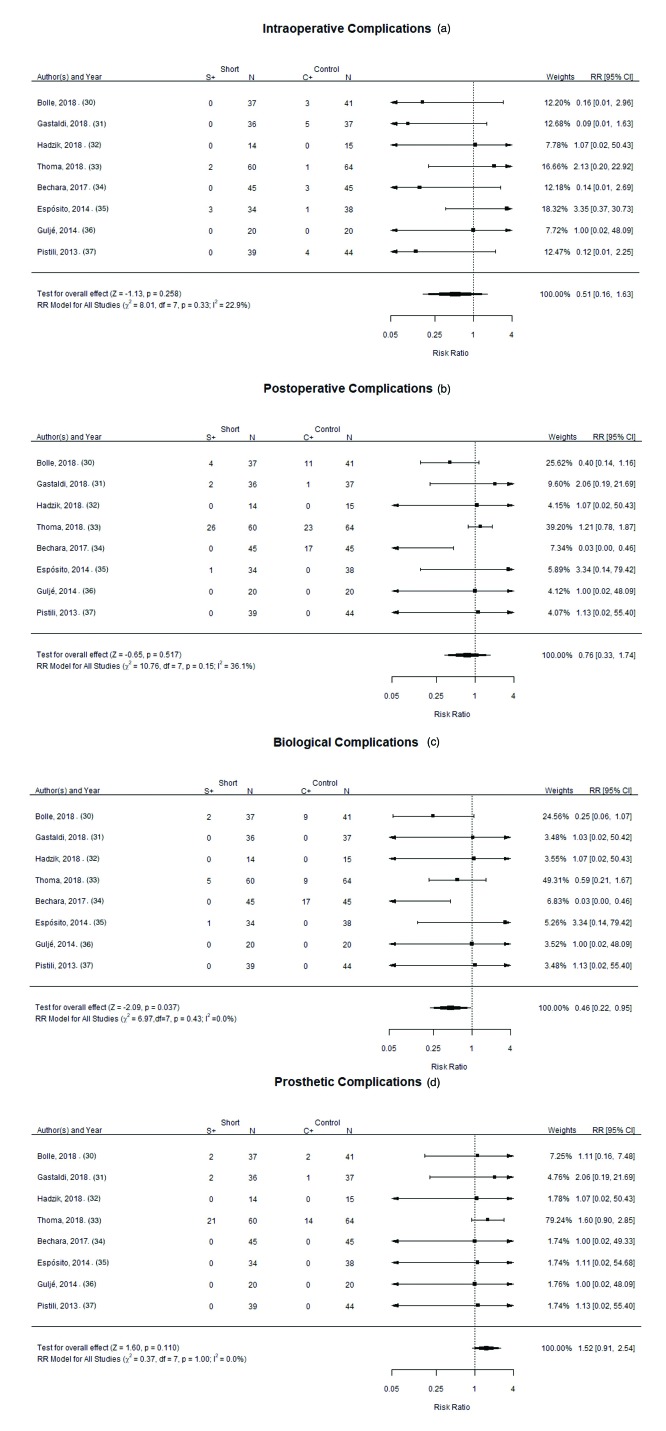
Forest plot for the event:(a) “intra-operative complications”; (b) “post-operative complications”; (c) “biological complications”; (d) “prosthetic complications”.

Intra-operative complications:

The Chi2 test showed homogeneity between the studies (I2=22.9% *p*= 0.33); and the overall effect test found no statistically significant differences between control and study groups (RR of 0.51; 95% CI: [0.16-1.63]; *p*= 0.258), in relation to intra-operative complications (Fig. 3).

Post-operative complications:

The Chi2 test showed homogeneity between the studies (I2=36.1% *p*= 0.15); and the overall effect test found no statistically significant differences between control and study groups (RR of 0.76; 95% CI: [0.33-1.74]; *p*= 0.517), in relation to post-operative complications (Fig. 3).

Biological complications:

The Chi2 test did not find statistically significant heterogeneity between the studies (I2=0.0%; *p*=0.43). The overall effect test demonstrated that there were more biological complications in the control group (RR of 0.46; 95% CI: [0.22-0.95]; *p*=0.037) (Fig. 3).

Prosthetic complications

The Chi2 test demonstrated homogeneity between the studies (I2= 0.0% *p*=1.00); and the overall effect test didn’t find statistically significant differences between the control and study groups (*p*= 0.110). A high number of studies did not suffered any prosthetic complications, either for short implant groups or longer implant groups. A RR of 1.52; 95% CI: [0.91, 2.54] favored the control group slightly (Fig. 3).

## Discussion

Short implants are considered a reliable and predicTable alternative to bone augmentation procedures (7,11,13), reducing the rate of complications, intra-operative time, patient morbidity, and treatment costs (12). The fourth European Association for Osseointegration (EAO) consensus conference (31) reported a survival rate of 99.0% for short implants (<8 mm) after 16-18 months follow-up, considering their use a routine treatment. The present systematic review and meta-analysis defined the term ‘short’ to describe implants of less than 8 mm (<8 mm) in length in accordance with the definition proposed recently by Plonka *et al*. [2018] (11).

- Survival rate

The results of meta-analysis did not find statistically significant differences in survival rates between short implants (<8mm) and longer implants (≥8mm) with lateral sinus floor elevation. None of the RCTs analyzed reported statistically significant differences between control and test groups. Similar results were reported by Hadzik *et al*. (32), Guljé *et al*. (36) and *Pi*stilli *et al*. (37), who obtained 100% survival rates for both control and test implants. However, these results should be treated with caution because of the small numbers of failed implants in both groups and the short follow-up periods.

Short implants might be expected to suffer more failures than long implants after loading because of their bio-mechanical disadvantages. However, the results of the RCTs in this review did not demonstrate this effect. The high survival rate of short implants could be attributed to improved implant surfaces and connections. Traditionally, machined surface implants with external connections were used, but the development of internal connections and rough surfaces have increased the implants surface area, favoring bone-to-implant contact, reduced treatment time, implant diameter and length, so that they now produce similar or even better results in comparison with machined implants (38).

The length of the implants included in the study groups ranged between 4 mm and <8 mm. Anitua *et al*. (38) obtained a 98.2% survival rate for 114 extra-short implants (<6.5 mm) after a follow-up period of 26 months. Recently, Srinivasan *et al*. (39), 690 6-mm short implants were assessed, obtaining a cumulative survival rate of 93.7% for maxillary implants and 98.6% for maxillary and mandibular implants together.

Furthermore, in a retrospective study published by Tetsch *et al*. (11) showed implants of ≥10 mm with lateral sinus floor elevation had 98.3% implant survival rate after 15.5 years of follow-up.

- Complications

In reference to complications, two studies (30,34) reported statistically significant differences in favor of short implants, although the random-effects model showed statistically significant heterogeneity between studies. For this reason, complications were divided into four groups: 1) intra-operative complications; 2) post-operative complications: 2a) biological complications; and 2b) prosthetic complications. When complications were divided into subgroups, only Bechara *et al*. (34) reported a significantly higher number of post-operative biological complications in the control group. The complications associated with longer implants with lateral maxillary sinus augmentation were, in order of frequency: pain/swelling > sinus membrane perforation > nasal bleeding and post-operative headache > intra-operative bleeding > infection of the grafting material > migration of the implant into sinus maxillary sinus. In the group of short implants (<8 mm) the most frequent complications were: sinus membrane perforation > nasal bleeding > migration of the implant into sinus maxillary sinus.

The most common prosthetic complication in both groups was screw loosening/fracture. In brief, incidence was slightly higher in study groups (short implants) although the difference was not statistically significant. Only a few studies reported prosthetic complications, comparing study groups with control groups, but the higher number of implants in the control groups suggest that longer implants (>8mm) with lateral sinus floor elevation suffered fewer prosthetic complications.

- Marginal Bone Loss (MBL)

In the present study, MBL in patients receiving longer implants (≥8mm) with lateral sinus floor elevation was statistically higher compared with patients who received short implants (<8mm).

This results can be justified by the article of Galindo-Moreno *et al*. (40), evaluated the MBL of implants placed in native bone or in grafted sinus lift in the maxilla. Concluded that “implants placed in sites that received maxillary sinus augmentation exhibited more marginal bone loss than implants placed in pristine bone, although marginal bone loss mainly occurred during the first 12 months after functional loading”.

In the RCT by Bechara *et al*. (34), the study group included short implants (6 mm in length) placed in healed sites and post-extraction sockets; however, no statistically significant differences in MBL between the two groups were found at either 1- or 3-year follow ups.

Due to the heterogeneity of the publications reviewed and the lack of information this systematic review suffered some limitations. Three out eight reviewed publications were considered with high risk of bias what might affect the obtained results. Furthermore, it was not possible to draw any definitive conclusions regarding the success rate, impact of implant diameter, implant design, and the type of prosthetic restoration on the variables investigated. Little information was available in the studies reviewed regarding the type of prosthetic reconstruction (single unit or multiple units), number of prosthetic reconstructions, and the number of implants per prosthetic unit. Moreover, the outcomes of the present review should be interpreted with caution given the small sample size and short follow-up times of the studies analyzed. In addition, five (30,33-35,37) out the 8 papers analyzed were published by the same research team.

## Conclusions

Within the limitations of the present systematic review, prosthetic rehabilitations with short implants (<8mm) in the maxillary posterior areas are a predicTable treatment option as an alternative to sinus floor elevation No statistically significant differences in the survival rate and complications were found between short (<8mm) and longer implants (≥8mm) with lateral sinus floor elevation. Nevertheless, longer implants (≥8mm) in combination with lateral sinus elevation presented significantly greater marginal bone loss.
